# Analysis of Prostate-Specific Antigen Transcripts in Chimpanzees, Cynomolgus Monkeys, Baboons, and African Green Monkeys

**DOI:** 10.1371/journal.pone.0094522

**Published:** 2014-04-14

**Authors:** James N. Mubiru, Alice S. Yang, Christian Olsen, Sudhir Nayak, Carolina B. Livi, Edward J. Dick, Michael Owston, Magdalena Garcia-Forey, Robert E. Shade, Jeffrey Rogers

**Affiliations:** 1 Southwest National Primate Research Center, Texas Biomedical Research Institute, San Antonio, Texas, United States of America; 2 Department of Biology, St. Mary's University, San Antonio, Texas, United States of America; 3 Biomatters Inc., Newark, New Jersey, United States of America; 4 Department of Biology, The College of New Jersey, Ewing, New Jersey, United States of America; 5 Department of Molecular Medicine and Institute of Biotechnology, University of Texas Health Science Center, San Antonio, Texas, United States of America; 6 Human Genome Sequencing Center, Baylor College of Medicine, Houston, Texas, United States of America; University of Oxford, United Kingdom

## Abstract

The function of prostate-specific antigen (PSA) is to liquefy the semen coagulum so that the released sperm can fuse with the ovum. Fifteen spliced variants of the *PSA* gene have been reported in humans, but little is known about alternative splicing in nonhuman primates. Positive selection has been reported in sex- and reproductive-related genes from sea urchins to *Drosophila* to humans; however, there are few studies of adaptive evolution of the *PSA* gene. Here, using polymerase chain reaction (PCR) product cloning and sequencing, we study *PSA* transcript variant heterogeneity in the prostates of chimpanzees (*Pan troglodytes*), cynomolgus monkeys (*Macaca fascicularis*), baboons *(Papio hamadryas anubis),* and African green monkeys *(Chlorocebus aethiops)*. Six *PSA* variants were identified in the chimpanzee prostate, but only two variants were found in cynomolgus monkeys, baboons, and African green monkeys. In the chimpanzee the full-length transcript is expressed at the same magnitude as the transcripts that retain intron 3. We have found previously unidentified splice variants of the *PSA* gene, some of which might be linked to disease conditions. Selection on the *PSA* gene was studied in 11 primate species by computational methods using the sequences reported here for African green monkey, cynomolgus monkey, baboon, and chimpanzee and other sequences available in public databases. A codon-based analysis (dN/dS) of the *PSA* gene identified potential adaptive evolution at five residue sites (Arg45, Lys70, Gln144, Pro189, and Thr203).

## Introduction

Prostate-specific antigen (PSA) is encoded by the kallikrein-3 (*KLK3*) gene, which belongs to the tissue kallikrein (*KLK*) gene family; the official name of the gene is *KLK3*, although it is commonly referred to as *PSA* [1], [2]. The *PSA* gene is composed of five exons and four introns. Part of the first exon codes for a signal peptide that targets the protein for secretion [3], [4]. In the PSA protein, residues His57, Asp102, and Ser195 have been reported to be important for proteolytic activity of PSA and other kallikreins, and they are commonly referred to as the catalytic triad [5], [6]. PSA has an activation peptide of seven amino acids that is cleaved by KLK2 protein to generate enzymatically active PSA [7], [8]. PSA is highly expressed by prostatic epithelial cells and is abundant in seminal plasma [9]. After ejaculation, semen forms a coagulum through the linkage of semenogelin proteins [10], [11]. Then, the coagulum has to be liquefied so that the sperm can be released and fuse with the ovum. In humans, great apes, and Old World monkeys this is done by PSA [3], [11]–[14].

Alternative splicing is a common mechanism in nature to enhance protein diversity, and it occurs in more than 90% of human genes [15], [16]. Alternative splicing of the *PSA* gene produces at least 15 transcripts of 0.7–6.1 kb [17].

Positive selection has been reported in sex- and reproductive-related genes from sea urchins to *Drosophila* to humans [12], [18], [19]. However, there is scant data regarding the *PSA* gene despite its importance in reproductive success. Marques et al. (2012) recently provided evidence of adaptive evolution of the *KLK3* gene toward an expanded enzyme spectrum that has an effect on the hydrolysis of semen coagulum [20]. In another study that analyzed the *PSA* gene, Clark and Swanson (2005) reported an overall dN/dS value of 0.38 for the *PSA* gene in human-chimpanzee pairwise comparisons, suggesting purifying selection [21].

The aims of this study are to investigate *PSA* transcript heterogeneity in four nonhuman primates commonly used in biomedical research and to study the mode of selection exerted on this gene in primates. Our previous data showed that cynomolgus monkeys have high serum PSA concentrations compared to baboons, which have low concentrations [22]. The chimpanzee was examined because of its close evolutionary relationship to humans.

## Materials and Methods

### Ethics Statement

We opportunistically sampled animals that presented for necropsy. Six cynomolgus monkeys (*Macaca fascicularis*) and baboons (*Papio hamadryas anubis*) and four chimpanzees (*Pan troglodytes*) were from the Southwest National Primate Research Center (SNPRC), located at the Texas Biomedical Research Institute in San Antonio, Texas. Three African green monkey (*Chlorocebus aethiops*) samples were from the Department of Pathology/Comparative Medicine, Wake Forest University of Health Sciences, Winston-Salem, North Carolina. The chimpanzees died of heart failure as a result of chronic cardiomyopathy. Three cynomolgus monkeys were removed from the colony and euthanized because of positive titers to simian retrovirus. One baboon with a sperm granuloma that tested positive for herpes was removed from the colony and euthanized. The other animals were part of research studies and were euthanized at the end of the studies.

Animals were chemically restrained with an injection of ketamine HCl and euthanized humanely under the supervision of a veterinarian using euthanasia solution (Fatal plus, Vortech Pharmaceuticals, Dearborn, MI). Confirmation of death was determined by monitoring for absence of pulse, respiration, and neural reflexes. The Institutional Animal Care and Use Committee of the Texas Biomedical Research Institute approved all procedures. Human prostate cDNAs were from cryopreserved human prostate tissues that were collected following radical prostatectomies under an Institutional Review Board approved protocol for the Department of Pathology at the University of Texas Health Science Center, San Antonio, and have been described previously [23].

### Transcript Cloning

We extracted total RNA from prostates using Trizol reagent (Life Technologies, Carlsbad, CA), reverse-transcribed them, and performed polymerase chain reaction (PCR) analysis, as described previously [22].

Primer sequences were based on the cynomolgus monkey and chimpanzee sequences (GenBank accession numbers AY647976, NM_001009136) [12], [24] and are shown in Tables 1 and 2. The forward primers were located in exon 1, and the reverse primers were located in exon 5 or in intron 3. We used primer sequences located in intron 3 because of reports indicating that some *PSA* variants contain sequences in the third intron [25]. To obtain the complete coding sequences, we cloned the PCR products into the pCR2.1-TOPO vector (Life Technologies, Carlsbad, CA) and sequenced them. Cloning using the two primer sets was done at least four times for each animal species. Each time 22 colonies were picked and cultured. Positive clones were characterized by restriction enzyme analysis according to their size on an agarose gel. One clone of each size was sequenced. We predicted the presence of a signal peptide to determine whether the transcripts could be secreted outside the cell using the Signal Peptide Prediction website (http://www.cbs.dtu.dk/services/SignalP/).

**Table 1:** Primer sequences used for cloning cynomolgus monkey, baboon, African green monkey, and chimpanzee *PSA*.

Primer	Sequence 5′–3′	Location of Primer	Product Size
Cynomolgus monkey, baboon, and African green monkey full-length *PSA* cDNA cloning			
PSA outer forward (A-07-2911)	5′-CTGGACAGCTGTGTCACCAT-3′	Exon 1	1081 bases
PSA outer reverse (A-07-3585)	5′-TGAGATGTCTCCAGGCATGA-3′	Exon 5	
PSA inner forward (A-07-2910)	5′-CTGTGTCACCATGTGGGTTC-3′	Exon 1	811 bases
PSA inner reverse (A-07-2908)	5′-GAGTTGATGGGGTGCTCAG-3′	Exon 5	
Chimpanzee full-length *PSA* cDNA cloning			
Chimp outer forward (12-3746)	5′-CTGTGTCACCATGTGGGTCC-3′	Exon 1	847 bases
Chimp outer reverse (12-3747)	5′-GGTCATTTCCAAGGTTCCAA-3′	Exon 5	
Chimp inner forward (A-11-1635)	5′-ACCATGTGGGTCCTGGTTGT-3′	Exon 1	808 bases
Chimp inner reverse (12-3748)	5′-AGGGAGTTGATAGGGGTGCT-3′	Exon 5	
*PSA* cDNA cloning of transcripts that use intron 3 (all four species)			
Chimp outer forward (A-07-2910)	5′-CTGTGTCACCATGTGGGTTC-3′	Exon 1	568 bases
Chimp outer reverse (A-11-1472)	5′-CTGTCCCCTCCTCCCTCAG-3′	Intron 3	
Chimp inner forward (A-11-1635)	5′-ACCATGTGGGTCCTGGTTGT-3′	Intron 1	568 bases
Chimp inner reverse (A-11-1636)	5′-CCTCAGACCCAGGCATCT-3′	Intron 3	

**Table 2:** Primer sequences used for real-time quantification of chimpanzee full-length and short-version *PSA* transcripts.

Primer	Sequence 5′–3′	Location of Primer
Chimp full-length forward	5′-GCAAGTTCACCCTCAGAAG-3′	Exon 4
Chimp full-length reverse	5′-GAAGCACACCATTACAGACAAG-3′	Exon 5
Chimp full-length probe	5′-CCCCCAGAATCACCCGAGCAGG-3′	Exon 4–exon 5 junction
Chimp short-length forward	5′-GGATGCTGTGAAGGTCATGG-3′	Exon 3
Chimp short-length reverse	5′-CATACACTCCTCTGGTTCGATG-3′	Exon 3–intron 3 junction
Chimp short-length probe	5′-AGTGCTGGCTCCTGGGTGG-3′	Exon 3

### Real-Time PCR

A closer look at the transcripts indicated that they could be broadly grouped into those that used the sequences in intron 3 and those that did not. We performed real-time PCR to explore whether expression of transcripts that do not use intronic sequences takes precedence over those that do. We carried out quantitative reverse transcription (RT) PCR using TaqMan technology (Life Technologies, Carlsbad, CA). We designed primers and probes for the quantitation of the chimpanzee full-length and short-version transcripts using the IDTDNA SciTools software (Integrated DNA Technologies Inc., Coralville, IA); the sequences are shown in Table 2. TaqMan 18s ribosomal RNA probe and primers served as the internal control. All samples and standards were analyzed in triplicate. We conducted real-time fluorescence-based PCR using the ABI Prism 7900 real-time PCR thermal cycler (Applied Biosystems, Foster City, CA) under vendor-specified conditions. We determined the values of the unknown samples from the standard curves prepared in each assay and normalized the concentration of the *PSA* transcripts to 18s ribosomal RNA.

### Analysis of Selection

We assessed the evolutionary selection acting on the *PSA* gene using 11 *PSA* gene sequences. Our group produced four sequences, one each for *Pan troglodytes*, *Macaca fascicularis*, *Papio hamadryas anubis*, and *Chlorocebus aethiops*; these sequences are reported in this paper. Eight other sequences—*Macaca fascicularis* (AY647976), *Macaca mulatta* (NM_001042776.1), *Macaca fuscata* (KC155626.1), *Homo sapiens* (NM_001648.2), *Pan paniscus* (DQ150478.1), *Gorilla gorilla* (KC155627.1), *Pongo pygmaeus* (DQ150480.1), and *Nomascus leucogenys* (UCSC genome Browser, Gibbon chr10)—were retrieved from public databases.

Protein sequences were aligned using the Multiple Sequence Comparison by Log-Expectation (MUSCLE) program, and codon-guided alignments were generated using Java Codon Delimited Alignment (JCoDA) [26], [27]. Preliminary analysis of dN/dS (nonsynonymous changes/synonymous changes) was performed on all pairwise comparisons using the coding region and sliding window (window 20, jump 5) analysis in JCoDA. Although purifying selection dominated across all comparisons, multiple regions in the C-terminal region appeared to be under relaxed or positive selection or with dN/dS (ω) values greater than 2. To identify individual residues under positive selection, we used the Phylogenetic Analysis by Maximum Likelihood (PAML) software package, version 4.7, with the following comparisons: between null hypothesis M0 (one ratio for all branches) and hypothesis M3 (discrete), between hypotheses M1 (neutral) and M2 (selection), and between hypotheses M7 (β-distributed 0 to 1) and M8 (β-distributed and estimated ω). Sites under selection were identified using the Bayes empirical Bayes (BEB) approach, the naive empirical Bayes (NEB) approach, and the likelihood ratio test (LRT) in CODEML (part of the PAML package) [28]–[31].

Phylogenetic trees were constructed using neighbor-joining, maximum parsimony, maximum-likelihood, and unweighted pair group method with arithmetic mean (UPGMA) methods provided with the Phylip package, version 3.695 [32]. In each case, tree topology was tested using bootstrapping with 100 replicates.

We were cognizant of the reported chimeric nature of the *KLK2*/*KLK3* genes in both *Gorilla gorilla* and *Nomascus leucogenys*. Marques et al. (2012) indicated that the gorilla and gibbon *KLK3* gene is in fact a fusion of *KLK3* and *KLK2* (*cKLK*), in which the first four exons of the fused gene are orthologous to *KLK3* and the last exon is more similar to *KLK2* [20]; therefore in our analysis we excluded exon 5. However, it has also been reported that at the protein level these genomic rearrangements in exon 5 account for only minor amino acid replacements and are not predicted to alter protein structure and function and that *cKLK* is likely a functional *KLK3*-like gene [20].

When numbering the amino acid residues, we followed both the PSA protein numbering and also the convention used for serine proteases. The convention numbering system is based on chymotrypsin and denotes that the catalytic triad is composed of His57, Asp102, and Ser195 and that the “Kallikrein loop” is composed of an 11 amino acid insertion (95A–95K) that is specific for *PSA* [33].

## Results

### Transcript Cloning

**Figure 1:**
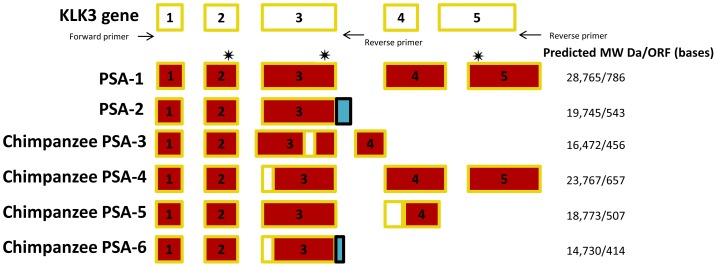
Structure of the PSA variants identified in cynomolgus monkey, baboon, African green monkey, and chimpanzee prostates. The retained intronic sequences are shown in blue, and the missing parts of exons are shown by blank boxes. The molecular weight (MW) and open reading frame (ORF) length of each predicted translation product were determined using the computational tool located at the EXPASY website (http://expasy.org/) and are shown to the right of the corresponding transcript. The residues that make up the catalytic triad are shown by asterisks.

**Figure 2:**
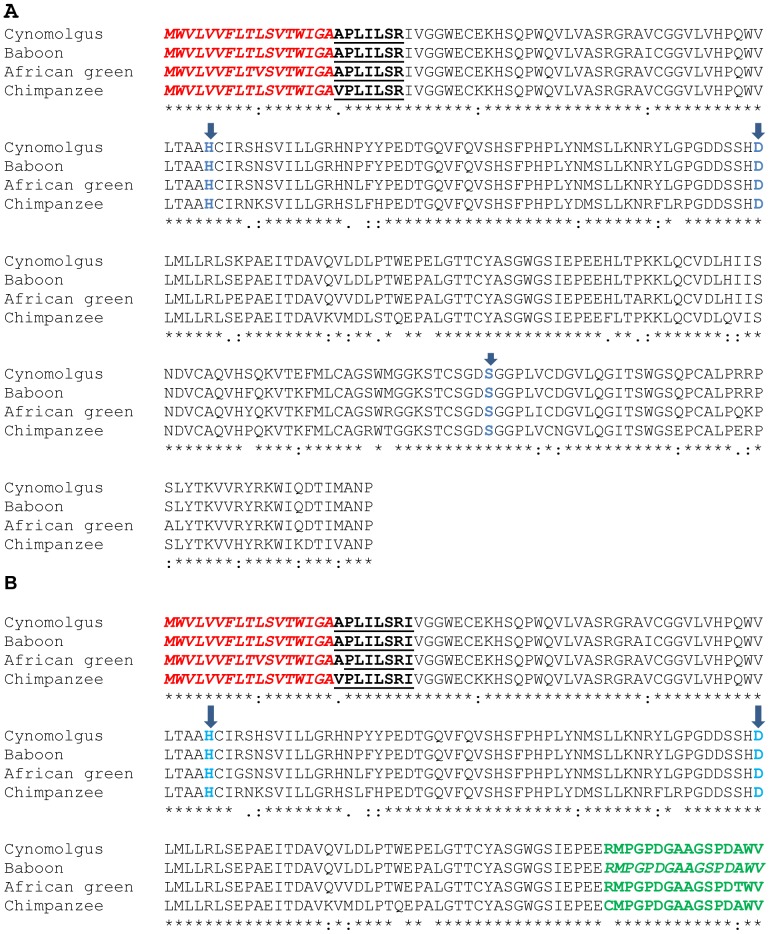
Alignment of the predicted amino acid sequences of the PSA-1 transcripts (panel A) and PSA-2 transcripts (panel B). The signal peptide is indicated in red italics. The residues that make up the catalytic triad are in blue and are indicated by arrows. The activation sequence is in bold and underlined. The unique 16-residue sequence that emanates from use of intron 3 is shown in green bold italic. The alignments were carried out using the CLUTALW program available at http://www.ebi.ac.uk/toolsmsa/clustalw2/ (Thompson et al., 1994 [41]).

We identified two *PSA* transcripts in the cynomolgus monkey, baboon, and African green monkey and six *PSA* transcripts in the chimpanzee prostate (Table 3; Figure 1). From all four species the same two main transcripts were identified: *PSA-1* has the five exons characteristic of the kallikrein (*KLK*) gene family, and *PSA-2* is missing exons 4 and 5 and uses sequences in intron 3. Because of intron retention, a unique 50-base sequence is added to exon 3. The *PSA-1* transcript is derived from five exons with an open reading frame of 786 bases encoding a 261 amino acid protein and has a predicted molecular weight of 28,839 Da (Figure 2). This sequence is orthologous to the human *KLK3* transcript variant 1 (NM_001648.2). The cynomolgus monkey *PSA-1* is identical to the one reported by Marshal et al. (2006) under GenBank accession number AY647976.1 [24]. The baboon sequence is identical to sequences under GenBank accession numbers NM_001112745, DQ150485 [21], and EF676031 [22]. The chimpanzee sequence is identical to a sequence under GenBank accession number DQ150477 [21]. The African green monkey *PSA-1* is novel, because no similar transcript was found in the National Center for Biotechnology Information (NCBI) GenBank.

**Table 3:** Summary of *PSA* gene splice events in chimpanzee (*Pan troglodytes*), cynomolgus monkeys (*Macaca fascicularis*), baboons (*Papio hamadryas anubis*), and African green monkeys (*Chlorocebus aethiops*).

	*PSA* Variant	Splicing Event
Chimpanzee (6 transcripts)	Chimpanzee *PSA-1*	Complete 5 exons
	Chimpanzee *PSA-2*	Uses sequences in intron 3
	Chimpanzee *PSA-3*	Skips 89 bases in exon 3
	Chimpanzee *PSA-4*	Skips 129 bases in exon 3
	Chimpanzee *PSA-5*	Skips 36 bases in exon 4
	Chimpanzee *PSA-6*	Skips 129 bases in exon 3 and uses sequences in intron 3
Cynomolgus monkey (2 transcripts)	Cynomolgus *PSA-1*	Complete 5 exons
	Cynomolgus *PSA-2*	Uses sequences in intron 3
Baboon (2 transcripts)	Baboon *PSA-1*	Complete 5 exons
	Baboon *PSA-2*	Uses sequences in intron 3
African green monkey (2 transcripts)	African green monkey *PSA-1*	Complete 5 exons
	African green monkey *PSA-2*	Uses sequences in intron 3

The *PSA-2* transcript has an open reading frame of 543 bases, which encodes a 180 amino acid protein, and has a molecular weight of 19,820 Da (Figure 2). The chimpanzee transcript is identical to a chimpanzee sequence available in the GenBank under accession number NM_001009136.1 [12] and is orthologous to gorilla and orangutan sequences numbers AY781395.1 and AY78139.1, respectively. It is 98% similar to a human sequence identified as *PSA-RP2* reported by Heuzé-Vourc'h et al. (2001) (GenBank AJ310937, number CAC41631) [25]. The cynomolgus monkey, baboon, and African green monkey *PSA-2* sequences are novel; no similar transcripts were found in the NCBI GenBank.

Four more transcripts were identified in the chimpanzee. Chimpanzee *PSA-3* has an open reading frame of 456 bases. It skips 89 bases of exon 3, resulting in an early stop codon. It codes for a 151 amino acid protein and has a predicted molecular weight of 16,470 Da. It is novel, as no similar transcript was found in the NCBI GenBank.

Chimpanzee *PSA-4* is a result of an in-frame deletion of 129 bases of exon 3. This transcript has an open reading frame of 657 bases, which encodes a 218 amino acid protein, and has a molecular weight of 23,768 Da. This transcript is orthologous to the human *KLK3* transcript variant 4 (NCBI accession number NM_001030048) and a rhesus monkey sequence (UniProt number F6TBJ4_MACMU).

Chimpanzee *PSA-5* is novel, consisting of four exons. It skips 38 bases in the 5′ part of exon 4, resulting in an early stop codon, and is therefore shorter than chimpanzee *PSA-1*. It has an open reading frame of 507 bases and encodes a 168 amino acid protein and has a predicted molecular weight of 18,774. Da.

Chimpanzee *PSA-6* is also novel. It is similar to the chimpanzee *PSA-2* transcript, except that it skips 129 bases in the 5′ part of exon 3 as a result of use of an alternative splice acceptor site. It has an open reading frame of 414 bases, which encodes a 137 amino acid protein, and has a molecular weight of 14,730 Da.

We cannot rule out the possibility that other splice variants were not found by our cloning strategy, especially those expressed at much lower levels.

All the novel transcripts reported in this study for nonhuman primates were also found in human prostate cDNA, indicating greater similarity among the prostate transcriptomes across primates than has previously been appreciated. In addition, all the *PSA* transcripts reported have an intact signal peptide consisting of 17 amino acids. However, only the *PSA-1* transcripts have an intact catalytic triad.

A comparison of the amino acid sequences of the *PSA-1* and *PSA-2* transcripts is shown in Figure 2. The transcripts reported in this study have been deposited in the NCBI GenBank under accession numbers KC853005 (*Chlorocebus aethiops PSA-1*), KC853006 (*Chlorocebus aethiops PSA-2*), KC853007 (*Macaca fascicularis PSA-2*), KC853008 (*Papio anubis PSA-2*), KC853009 (*Pan troglodytes PSA-3*), KC853010 (*Pan troglodytes PSA-4*), KC853011 (*Pan troglodytes PSA-5*), and JX445923 (*Pan troglodytes PSA-6*).

### Real-Time PCR

**Figure 3:**
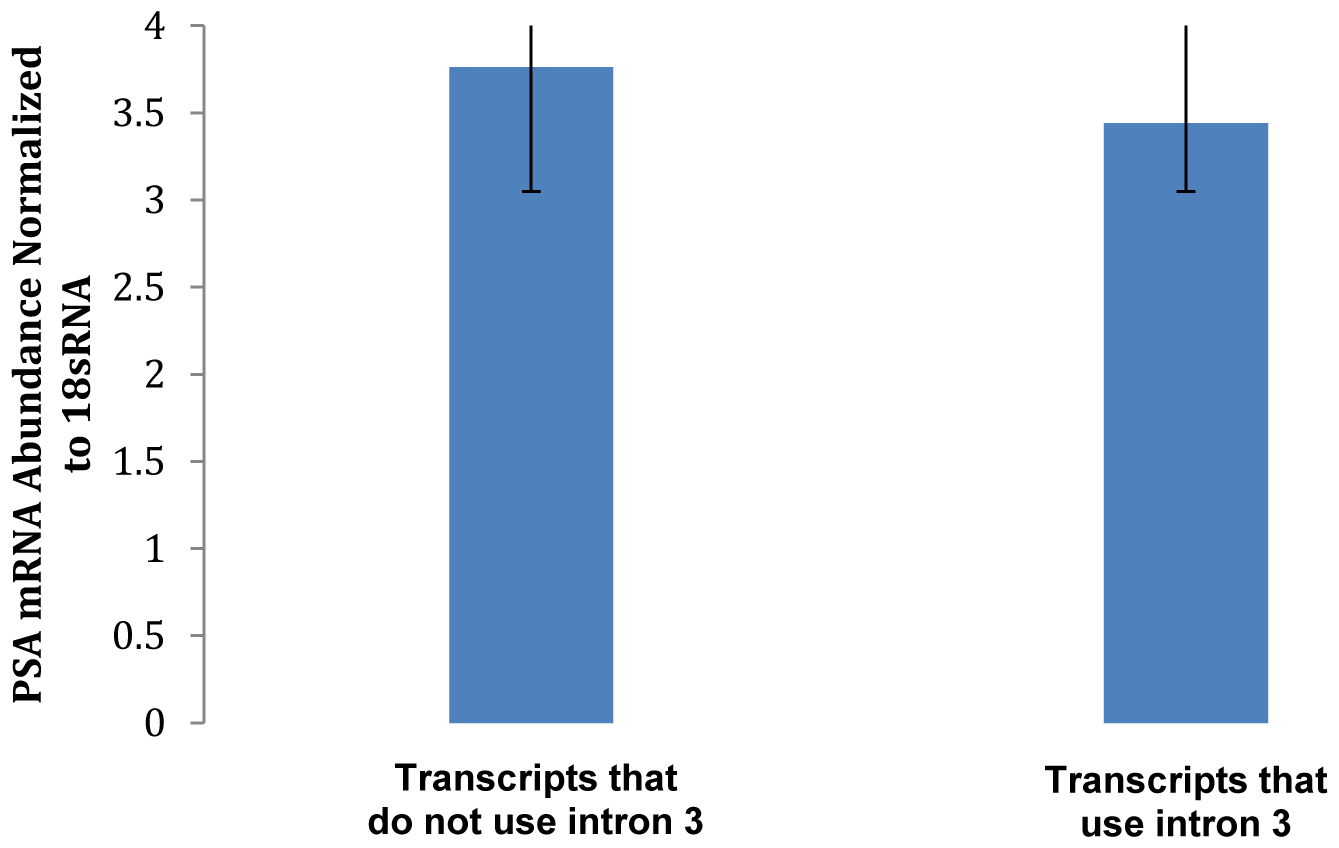
Relative real-time PCR quantitation of chimpanzee transcripts that use intron 3 sequences compared to those that do not (mean ± standard deviation, *n* = 3). Individual tissue samples were reverse-transcribed and *PSA* transcript mRNA was quantitated using the primer/probe sets described in Table 2. Relative levels of the *PSA* variants were normalized to the 18s RNA levels in the same samples.

We next investigated quantitative differences in expression levels among the six chimpanzee transcripts. Using real-time PCR techniques, we did not find a significant difference in amplification rates between the transcripts that use sequences in intron 3 (chimpanzee *PSA-2* and *PSA-6*) from those that do not (chimpanzee *PSA-1*, *-3*, *-4*, *-5*) (Figure 3).

### Phylogenetic Analysis

**Figure 4:**
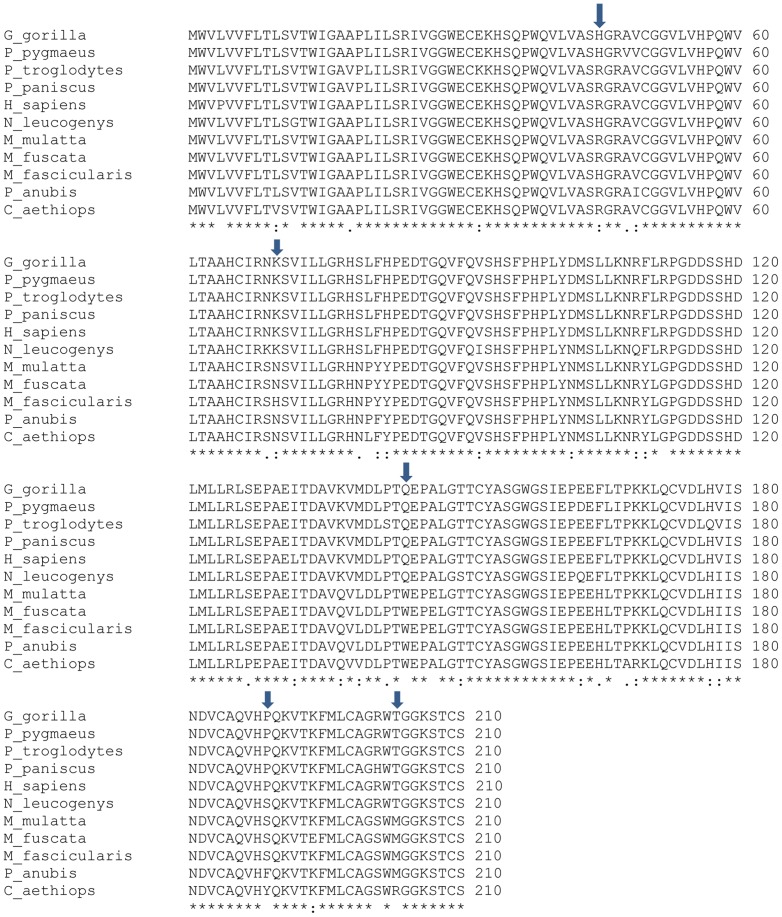
Multiple sequence comparison by log expectation (MUSCLE) program guided alignments of *PSA-1* transcripts from 11 species. The codons that are under positive selection are indicated by arrows. Fully conserved residues are indicated by asterisks, partly conserved residues by colons, and weakly conserved residues by periods. The species' complete scientific names are as follows: *Pan troglodytes*, *Pan paniscus*, *Homo sapiens*, *Pongo pygmaeus*, *Gorilla gorilla*, *Nomascus leucogenys*, *Macaca fascicularis*, *Macaca fuscata*, *Macaca mulatta*, *Papio hamadryas anubis*, and *Chlorocebus aethiops*.

We compared the *PSA* gene sequences among 11 primate species to analyze selection on the *PSA* gene. The pairwise dN/dS values across all codon sites of the *PSA* gene of any two species did not exceed the threshold of 1, indicating purifying selection (Table S1). Although purifying selection dominated across all comparisons, multiple regions in the C-terminal region appeared to be under relaxed or positive selection with dN/dS (ω) values greater than 2. To identify individual residues under positive selection, we used the PAML software package, version 4.7, with the following comparisons: M0 vs. M3, M1 vs. M2, and M7 vs. M8 using the BEB and NEB approaches and the likelihood ratio test. All model comparisons with CODEML identified multiple residues with *p*>0.5 (probability of selection greater than 0.5), indicating directional selection. The M7 vs. M8 comparisons using the NEB and BEB approaches were similar, identifying five codons with dN/dS values greater than 2 (Table 4; Figure 4). When the codon-specific dN/dS value data were compared with the alternative splicing data, we found that several splice variants originated at codons with dN/dS values greater than 2 (Table 4).

**Table 4:** Bayes empirical Bayes likelihood ratio test of *PSA* gene codons that are under positive selection using model M7 vs. model M8.

Position and Residue Change (*KLK3* Numbering)	Position and Residue Change (Chymotrypsin Numbering)	dN/dS	Probability of dN/dS >1	Splice Variant Generated at Site
H45R	H37R	4.40*	0.98	
N69K/S	N61K/S	2.64	0.6	Chimpanzee *PSA-6*, Chimpanzee *PSA-4*
Q144W	Q128W	2.60	0.60	
P189S	P173S	4.30	0.94	
T203M/R	T187M/R	3.51	0.77	

* *p*<0.05.

**Figure 5:**
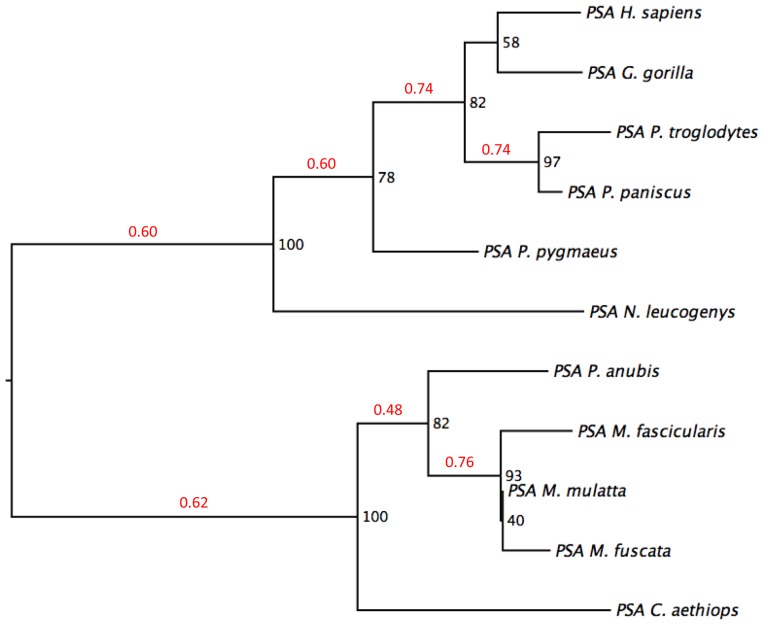
Phylogenetic tree constructed using neighbor-joining, maximum parsimony, maximum-likelihood, and unweighted pair group method with arithmetic mean (UPGMA) methods provided in the Phylip package, version 3.695. The numbers at each node are bootstraps, and the numbers in red on top of each branch are dN/dS values for that branch.

All methods generated similar consensus trees with similar bootstrap support for internal nodes. The only exception was the branching of *Gorilla gorilla* and *Nomascus leucogenys*, which varied between methods. The maximum-likelihood phylogenetic tree is shown in Figure 5, and the branching order is similar to the topology reported by others, except for the placement of *Gorilla gorilla* [20]. We looked at branch-specific variation in selective pressure along phylogenetic lineages. This did not reveal any statistically significant differences, but we noted that the variations in dN/dS values ranged from 0 to 0.76 (Figure 5).

## Discussion

We found six alternatively spliced *PSA* variants in the chimpanzee but only two in the cynomolgus monkey, baboon, and African green monkey. To the best of our knowledge, this study is the first to report the African green monkey *PSA* mRNA sequence and also the first to report on alternative splicing of the *PSA* gene in nonhuman primates. It is important to note that the identification of alternative splice variants depends on the primers used, and therefore we cannot rule out the possibility that there are other splice variants not found by our cloning primers.

Alternative splicing of the *PSA* gene in this study involved exon truncation, intron retention, or a combination of two splicing events. In a study of human kallikrein genes, Kurlender et al. (2005) found exon skipping to be the most common splicing event, with internal exon deletion being less common in kallikrein genes [34]. However, in our study we found retention of sequences in intron 3 to be the most common splicing event; it was found in all the species studied. Real-time PCR data indicated that in chimpanzees the expression of transcripts that do not use sequences in intron 3 did not take precedence over those that do, although we cannot rule out the possibility that under certain physiological conditions there might be differences in transcript expression. It is not clear why alternative splicing of the *PSA* gene is more common in chimpanzees than in cynomolgus monkeys, baboons, and African green monkeys, but chimpanzees do share a more recent common ancestor with humans than any other nonhuman primates do. All the transcripts reported here have orthologous human counterparts. The *PSA-2* sequences are orthologous to the human *PSA-RP2* sequence, which has been shown to be up-regulated in prostate cancer [35], indicating potential links of these transcripts to prostatic diseases. The link between these transcripts and human disease conditions needs further study. It is important to note that the splice variants were found in areas of the gene that are likely to be under selective pressure.

Not surprisingly, given the role of PSA, purifying selection dominates in all pairwise comparisons. Further analysis using codon-based models in CODEML identified multiple residues in the *PSA* gene having dN/dS values greater than 1, indicating directional selection. These data suggest that selective pressure in the *PSA* gene is not uniform across all codon sites but rather is focused on specific regions. Five codons were found to have dN/dS values greater than 2, although only one His45 reached the 95% confidence level. It is important to note that another study also found codons 45, 189, and 203 to be positively selected [20]. In addition, we note that limiting our analysis to exons 1–4 curtailed our statistical power. When the same analyses were performed using full-length sequences with all five exons, we found additional codons that had dN/dS values greater than 1. Limiting our analyses to the first four exons was due to the reported fusion of *KLK3* and *KLK2* in *Gorilla gorilla* and *Nomascus leucogenys*, in which the first four exons of the fused gene are orthologous to *KLK3* and the last exon is more similar to *KLK2* [20].

At codon position 189 the amino acid proline is substituted for serine or phenylalamine. Substitution of proline residues in proteins is of significant interest because of proline's unique structural and functional properties. Proline has a rigid conformation with low flexibility; it can often be found in tight turns in protein structures and also introduces kinks into alpha helices because it is unable to adopt a normal helical conformation. It is likely that proline substitution at codon 189 results in secondary and tertiary structural alteration, leading to significant functional changes. Although we did not explore functional consequences of the change at codon position 189 in this study, this is an excellent question for future analysis.

Our results of codon-based selection indicate that dN/dS values for the *PSA* gene are low relative to other proteins from the seminal fluid and prostasome. Clark and Swanson (2005) reported a dN/dS value of 2.9 for β-microseminoprotein and a value of 14 for prostate-specific transglutaminase for the class of codons identified to be under positive selection [21]. It is possible that the critical role for the PSA protein in copulatory plug dissolution constrains the changes tolerated and maintains low dN/dS values. Alternatively, the atypical evolutionary history of the *PSA* gene (e.g., fusion of *KLK3* and *KLK2* in some lineages) may be playing a contributing role that has yet to be defined.

Previous studies have indicated that chimpanzees have low serum PSA concentrations [36]. In our unpublished data we also found that chimpanzee PSA concentrations range from 0.01 to 0.031 ng/ml, well below levels reported in humans and other nonhuman primate species [22]. We propose that *PSA* gene evolution in the chimpanzee lineage might have functional implications. Clark and Swanson (2005) also showed that although the *PSA* gene does not have high pairwise human-chimpanzee dN/dS values, it does show significant variation in selective pressure during its evolution and dN/dS values along some phylogenetic lineages exceeded 1 [21].

The placement of the gorilla in our phylogenetic tree may be due to incomplete lineage sorting, whereby the genealogy relating humans, gorillas, and chimpanzees varies across the genome and about 22% of all coding exons exhibit phylogenies that contradict the overall genomic phylogeny, in which humans are more similar to chimpanzees than to gorillas [37]. The genomic region containing *PSA* may be one of the regions that has a local phylogeny in which the gorilla sequences are indeed more similar to the human sequences than the chimpanzee sequences are to either human or gorilla.

Because the function of PSA is to liquefy the sperm coagulum formed after ejaculation, we speculate that the differences in alternative splicing reported in this study might be related to the mating systems of these species. The four species studied here (chimpanzees, cynomolgus monkeys, baboons, and African green monkeys) all exhibit multimale-multifemale mating systems in which any given adult female may mate with more than one male during a single estrous cycle. However, some data suggest that chimpanzee females may have larger average numbers of mates per cycle. A single chimpanzee female frequently mates with numerous males during a single estrous cycle [38], [39]. Multiple males may follow, compete for, and copulate with a female during a single cycle [39]. Such male-male competition also occurs in other species to some degree. But there is reason to believe that sperm competition is important in chimpanzees, as a rubbery and long-lasting “copulatory plug” that obstructs the sperm of subsequent mating events from accessing the ovum is produced by chimpanzee ejaculates and not by those of cynomolgus monkeys, African green monkeys, and baboons [40]. Further studies of transcript heterogeneity comparing several species that have different mating patterns would further elucidate the possible relationship between the alternative expression of the *PSA* gene and mating systems.

## Supporting Information

Table S1 **Species pairwise comparison of the dN/dS values of the** ***PSA*** **gene.**
(DOC)
