# Correction: Analysis of Prostate-Specific Antigen Transcripts in Chimpanzees, Cynomolgus Monkeys, Baboons, and African Green Monkeys

**DOI:** 10.1371/journal.pone.0106018

**Published:** 2014-08-15

**Authors:** The PLOS ONE Staff

The following information is missing from the Funding section: The study also used resources from the Department of Pathology/Comparative Medicine, Wake Forest University Health Science which were supported by NIH grant RR019963/OD010965 (PI: Jay R. Kaplan).
