# Supplementary material for: Analysis of Prostate-Specific Antigen Transcripts in Chimpanzees, Cynomolgus Monkeys, Baboons, and African Green Monkeys
Source: PLoS One. 2014 Apr 14;9(4):e94522. doi: 10.1371/journal.pone.0094522 (PMC3986117; doi:10.1371/journal.pone.0094522)
Supplement: Table S1 — Species pairwise comparison of the dN/dS values of the PSA gene. (DOC) [file pone.0094522.s001.doc]

**Table S1. Species pairwise comparison of the dN/dS values of the *PSA* gene.**

| **Species Name** | **Full Length (dN/dS)** | **Exons 1–4 (dN/dS)** |
| --- | --- | --- |
| *Gorilla gorilla* vs. *Chlorocebus aethiops* | 0.3969 | 0.3495 |
| *Gorilla gorilla* vs. *Papio hamadryas anubis* | 0.3654 | 0.3157 |
| *Gorilla gorilla* vs. *Pan troglodytes* | 0.2925 | 0.2648 |
| *Gorilla gorilla* vs. *Nomascus leucogenys* | 0.2009 | 0.2299 |
| *Gorilla gorilla* vs. *Macaca fascicularis* | 0.4122 | 0.3753 |
| *Gorilla gorilla* vs. *Macaca mulatta* | 0.4315 | 0.4014 |
| *Gorilla gorilla* vs. *Pongo pygmaeus* | 0.2428 | 0.1168 |
| *Gorilla gorilla* vs. *Pan paniscus* | 0.2381 | 0.1579 |
| *Gorilla gorilla* vs. *Macaca fuscata* | 0.4257 | 0.3946 |
| *Gorilla gorilla* vs. *Homo sapiens* | 0.3217 | 0.2397 |
| *Chlorocebus aethiops* vs. *Papio hamadryas anubis* | 0.4646 | 0.4058 |
| *Chlorocebus aethiops* vs. *Pan troglodytes* | 0.5014 | 0.4214 |
| *Chlorocebus aethiops* vs. *Nomascus leucogenys* | 0.4374 | 0.4154 |
| *Chlorocebus aethiops* vs. *Macaca fascicularis* | 0.5736 | 0.5431 |
| *Chlorocebus aethiops* vs. *Macaca mulatta* | 0.5668 | 0.5305 |
| *Chlorocebus aethiops* vs. *Pongo pygmaeus* | 0.7224 | 0.5814 |
| *Chlorocebus aethiops* vs. *Pan paniscus* | 0.4702 | 0.3865 |
| *Chlorocebus aethiops* vs. *Macaca fuscata* | 0.5385 | 0.4987 |
| *Chlorocebus aethiops* vs. *Homo sapiens* | 0.4958 | 0.3877 |
| *Pan hamadryas anubis* vs. *Pan troglodytes* | 0.4004 | 0.3832 |
| *Pan hamadryas anubis* vs. *Nomascus leucogenys* | 0.4014 | 0.3777 |
| *Pan hamadryas anubis* vs. *Macaca fascicularis* | 0.3891 | 0.3787 |
| *Pan hamadryas anubis* vs. *Macaca mulatta* | 0.3255 | 0.317 |
| *Pan hamadryas anubis* vs. *Pongo pygmaeus* | 0.5575 | 0.524 |
| *Pan hamadryas anubis* vs. *Pan paniscus* | 0.3730 | 0.3497 |
| *Pan hamadryas anubis* vs. *Macaca fuscata* | 0.3247 | 0.3161 |
| *Pan hamadryas anubis* vs. *Homo sapiens* | 0.3914 | 0.3508 |
| *Pan troglodytes* vs. *Nomascus leucogenys* | 0.3691 | 0.3962 |
| *Pan troglodytes* vs. *Macaca fascicularis* | 0.4920 | 0.4986 |
| *Pan troglodytes* vs. *Macaca mulatta* | 0.4872 | 0.4932 |
| *Pan troglodytes* vs. *Pongo pygmaeus* | 0.4865 | 0.5107 |
| *Pan troglodytes* vs. *Pan paniscus* | N/A | N/A |
| *Pan troglodytes* vs. *Macaca fuscata* | 0.4780 | 0.4806 |
| *Pan troglodytes* vs. *Homo sapiens* | 0.3913 | 0.4831 |
| *Nomascus leucogenys* vs. *Macaca fascicularis* | 0.4176 | 0.4 |
| *Nomascus leucogenys* vs. *Macaca mulatta* | 0.4363 | 0.4264 |
| *Nomascus leucogenys* vs. *Pongo pygmaeus* | 0.4277 | 0.4144 |
| *Nomascus leucogenys* vs. *Pan paniscus* | 0.3305 | 0.3374 |
| *Nomascus leucogenys* vs. *Macaca fuscata* | 0.4304 | 0.4183 |
| *Nomascus leucogenys* vs. *Homo sapiens* | 0.3534 | 0.3385 |
| *Macaca fascicularis* vs. *Macaca mulatta* | 0.6561 | 0.6405 |
| *Macaca fascicularis* vs. *Pongo pygmaeus* | 0.6729 | 0.6701 |
| *Macaca fascicularis* vs. *Pan paniscus* | 0.4602 | 0.4579 |
| *Macaca fascicularis* vs. *Macaca fuscata* | 0.4909 | 0.4789 |
| *Macaca fascicularis* vs. *Homo sapiens* | 0.4858 | 0.4594 |
| *Macaca mulatta* vs. *Pongo pygmaeus* | 0.7239 | 0.7397 |
| *Macaca mulatta* vs. *Pan paniscus* | 0.4539 | 0.45 |
| *Macaca mulatta* vs. *Macaca fuscata* | 0.3285 | 0.3208 |
| *Macaca mulatta* vs. *Homo sapiens* | 0.4802 | 0.4514 |
| *Pongo pygmaeus* vs. *Pan paniscus* | 0.3765 | 0.3806 |
| *Pongo pygmaeus* vs. *Macaca fuscata* | 0.6962 | 0.7007 |
| *Pongo pygmaeus* vs. *Homo sapiens* | 0.3223 | 0.2702 |
| *Pan paniscus* vs. *Macaca fuscata* | 0.4462 | 0.44 |
| *Pan paniscus* vs. *Homo sapiens* | 0.2595 | 0.3201 |
| *Macaca fuscata* vs. *Homo sapiens* | 0.4710 | 0.4414 |
